# Assessing for prenatal risk factors associated with infant neurologic morbidity using a multivariate analysis

**DOI:** 10.1038/s41372-023-01820-3

**Published:** 2023-11-10

**Authors:** Samhita Jain, Scott Oltman, Elizabeth Rogers, Kelli Ryckman, Mark Petersen, Rebecca J. Baer, Larry Rand, Xianhua Piao, Laura Jelliffe-Pawlowski

**Affiliations:** 1 Division of Neonatology, Department of Pediatrics, University of California, San Francisco, CA USA; 2 California Preterm Birth Initiative, University of California San Francisco, San Francisco, CA USA; 3 Department of Epidemiology & Biostatistics, University of California, San Francisco, CA USA; 4 Department of Epidemiology and Biostatistics, Indiana University Bloomington, Bloomington, IN USA; 5 Department of Pediatrics, University of California San Diego, La Jolla, CA USA; 6 Department of Obstetrics, Gynecology, and Reproductive Sciences, University of California San Francisco School of Medicine, San Francisco, CA USA; 7 Newborn Brain Research Institute, University of California, San Francisco, CA USA; 8 Weill Institute for Neuroscience, University of California, San Francisco, CA USA

**Keywords:** Risk factors, Cytokines

## Abstract

**Objective:**

To characterize the biochemical and demographic profiles of pregnant people with maternal immune activation (MIA) and identify the prenatal characteristics associated with neurologic morbidity in offspring.

**Study design:**

This was a retrospective cohort study of 602 mother-infant dyads with births between 2009 and 2010 in California. Multivariable logistic regression was used to build a MIA vulnerability profile including mid-pregnancy biochemical markers and maternal demographic characteristics, and its relationship with infant neurologic morbidity was examined.

**Results:**

Of the 602 mother-infant dyads, 80 mothers and 61 infants had diagnoses suggestive of MIA and neurologic morbidity, respectively. Our model, including two demographic and seven biochemical characteristics, identified mothers with MIA with good performance (AUC:0.814; 95% CI:0.7–0.8). Three demographic and five inflammatory markers together identified 80% of infants with neurological morbidity (AUC:0.802, 95% CI:0.7–0.8).

**Conclusion:**

Inflammatory environment in mothers with pre-existing risk factors like obesity, poverty, and prematurity renders offspring more susceptible to neurologic morbidities.

## Introduction

A growing body of evidence from epidemiologic and animal studies have implicated diverse maternal inflammatory conditions including infection [1, 2] and autoimmune disorders [3, 4] with increased risk of neurodevelopment disorders such as schizophrenia (SZ) [5–7], autism spectrum disorder (ASD) [8–11], cognitive delay [10], and bipolar disorder [12, 13] in the offspring. Population studies after the 1964 Influenza pandemic demonstrated a 2- and 3-fold increase in the risk of ASD [1] and SZ [12, 14, 15], respectively, in children born to mothers with influenza infection during pregnancy. More recently, results from the Swedish national health registry that included 1.7 million children reported that children born to mothers with an infection during pregnancy had a 79% increased risk of an autism diagnosis and a 24% increased risk of a depression diagnosis as adults [16]. Non-infectious etiologies of maternal immune activation (MIA) including maternal autoimmune disorders, allergies, asthma, acute stress, and exposure to environmental pollutants have also been linked to an enhanced risk of ASD and SZ [7, 17]. Lastly, results from a state-wide population-based cohort study (from our group) that included 3 million maternal infant dyads found that MIA was associated with an increased risk of preterm delivery and neurological morbidity including abnormal neurological exam, seizures, and periventricular leukomalacia in offspring as early as the neonatal period [18]. Taken together, these epidemiologic studies align with the MIA hypothesis suggesting that a maternal inflammatory environment during gestation may alter the trajectory of the developing fetal brain and increase the child’s susceptibility to a neurodevelopmental disorder.

Extensive research in animal models of MIA and some epidemiologic studies have underscored a critical role of cytokines in the pathophysiology of neurodevelopmental and psychiatric disorders. In fact, animal models have utilized interleukin (IL)-6, -17, or -2 to induce MIA and have demonstrated a link between these cytokines and several ASD and SZ-like behaviors in the exposed pups [9, 19, 20]. In studies with human subjects, evaluation of archived serum and amniotic fluid samples obtained from mothers of individuals with ASD have identified an association between various cytokines and chemokines with ASD or developmental delay in children [21–23]. Important to the present study is that most of these studies focused mainly on ASD as an outcome and analyzed a limited number of inflammatory molecules. Also, the effects of MIA on the infants’ neurologic outcomes are quite heterogeneous. Additionally, despite the increasing evidence associating MIA with increased risk of NDD in children, there are no objective guidelines that allow the identification of women with MIA.

For this study, we aimed to characterize the biochemical and demographic profile of pregnant women with MIA, to objectively identify mothers with immune activation during pregnancy. Further, we sought to build a vulnerability profile including maternal demographic and biochemical parameters to identify pregnancies most susceptible to infant neurologic morbidity.

## Methods

The mother-infant dyads in this study were drawn from the California Prediction of Poor Outcomes of Pregnancy (CPPOP) cohort [24, 25], which originates from a population-based dataset encompassing all singleton births in California occurring between July 2009 and December 2010. This dataset was established through the linkage of California birth certificate records with hospital discharge records by the California Department of Health Care Access and Information (HCAI) (available at https://hcai.ca.gov/). Within the CPPOP framework, these HCAI records were further linked to those of the California Genetic Disease Screening Program (GDSP) (available at https://www.cdph.ca.gov/Programs/CFH/DGDS/Pages/default.aspx), enabling the identification of two groups: 496 pregnancies with term births (≥37 weeks gestational age) and 496 with preterm births (<37 weeks gestational age). All of these pregnancies had undergone routine prenatal screening for aneuploidies and neural defects and after obtaining informed consent, the second trimester serum samples were made available for research purposes, sourced from the California Biobank Program (available at https://www.cdph.ca.gov/Programs/CFH/DGDS/Pages/cbp/default.aspx). As part of CPPOP efforts, from these 992 available banked serum samples, 602 maternal samples were randomly selected for serum metabolites testing and included 318 serum samples from pregnancies with term birth and 284 serum samples from pregnancies with preterm birth. This was a convenient random sample wherein total number was determined based on the financial resources available for testing. Thus, for this specific study, we included a total sample of 602 women with available serum samples, which had previously undergone immune marker testing as part of other CPPOP initiatives [24]. All cases and controls in this cohort possess demographic, characteristic, and diagnostic data accessible through linked hospital, birth, and death certificate records, covering the period from one year before infant birth to one year after, and from birth through the first year of life for infants. These records comprise diagnostic and procedure codes based on the International Classification of Diseases, 9th Revision, Clinical Modification (ICD-9) (available at https://www.cdc.gov/nchs/icd/icd9cm.htm).

This study utilized two primary composite outcomes: MIA, defined using ICD-9 codes for maternal infection, autoimmune disorder, allergy, asthma, atherosclerosis, or malignancy during pregnancy; and neurologic morbidity in the infant, defined using ICD-9 codes present in discharge records for the infant birth through one year of age for intraventricular hemorrhage (IVH), periventricular leukomalacia (PVL), seizures, abnormal neurologic examination, and abnormal neurologic imaging.

Demographic and clinical variables evaluated included maternal race/ethnicity, maternal age, maternal education, gestational age at delivery, trimester prenatal care began, adequacy of prenatal care, insurance type, participation in the women, infants, and children program (WIC), infant sex, prepregnancy body mass index (BMI), smoking status, parity, and interpregnancy interval (IPI). Adequacy of prenatal care was based on the Kotelchuck adequacy of prenatal care utilization index [26]. Insurance type was categorized into private, public as indicated by Medi-Cal status (California’s Medicaid), and “other” which included uninsured, other government insurance (military, tribal care), and self-pay. Insurance type, WIC participation, and maternal education all serve as proxies for socioeconomic status (SES), as income as a variable was unavailable.

Immune and growth-factor molecular testing was done using residual serum samples from second trimester (15–20 week) prenatal screening. Specimens were stored in 1 milliliter tubes at −80 °C. Markers tested included twenty interleukins, three interferons, eleven chemokine ligands, eight members of the tumor necrosis factor-alpha (TNFA) super family cytokines, twelve growth factors, three colony-stimulating factors, two soluble adhesion molecules, and leptin, plasminogen activator inhibitor-1 (PAI-1), resistin, and receptor for advanced glycosylation end products (RAGE) (Supplementary Table 1). Multiplex testing was performed by the Human Immune Monitoring Center (HIMC) at Stanford University (available at https://iti.stanford.edu/himc/immunoassays.html). The full panel of immune biomarkers was evaluated by this study given the complexity and interconnectedness of immune function particularly with regards to its substantial role in pregnancy [27, 28]. All markers were read using a Luminex 200 instrument (Austin, TX) in accordance with the manufacturer recommendations. Details regarding Luminex lab protocols at the HIMC are available on their website (https://iti.stanford.edu/himc/immunoassays.html). In summary, all markers were tested using a human multiplex kit that was purchased from Affymetrix Inc. (Santa Clara, CA) except for human soluble receptors, which were measured using a Millipore high sensitivity multiplex kit (HSCRMAG32KPX14) (Billerica, MA). Median fluorescence intensity (MFI) values were reported for all markers using Masterplex software (Hitashi Solutions, San Bruno, CA). MFI values were converted to pg/ml using an MFI average from two aliquots tested on the same plate. All inter-assay coefficients (CVs) were <15 % across all markers and all intra-assay CVs were <10%. Methods and protocols for the study were approved by the Committee for the Protection of Human Subjects within the Health and Human Services Agency of the State of California. This IRB approval prevents sharing individual level data (de-identified or not) with people who are not listed as investigators on the project and are protected by institutional firewall.

### Statistical analyses

To reduce skewness and minimize the influence of outliers, all biomarkers were natural log transformed from their raw values. Raw biomarker values were also categorized into quartiles where low (<25th percentile) and high (>75th percentile) concentrations were compared to intermediate (25–75th percentiles) concentrations. Crude analyses to compare infants with any mortality or major morbidity to healthy infants relied on *t*-tests and χ2 tests for continuous and categorical variables, respectively.

Multivariable logistic regression utilizing stepwise selection was used to create models for the composite outcomes of MIA and neurologic morbidity. All variables were permitted to enter the models with entry order determined by greatest significance and a *p*-value of <0.05 required to remain in the model. MIA was included as an independent variable within the neurologic morbidity model and to assess its importance, was both forced into the model and allowed to enter as described above. Model performance was assessed using area under the curve (AUC) from a receiver operating characteristic (ROC) curve wherein variable importance was evaluated using odds ratios (ORs) with 95% confidence intervals (95% CI). Cross-validation with replacement was used to assess MIA and neurologic morbidity model fit.

All analyses were performed using SAS 9.4 (SAS institute, Cary, NC). Methods and protocols for the study were approved by the Committee for the Protection of Human Subjects within the Health and Human Services Agency of the State of California and the Institutional Review Board of the University of California, San Francisco.

## Results

Within our sample of 602 mother-infant dyads, 80 (13.3%) pregnant women had a clinical diagnosis suggestive of MIA and 61 infants had a diagnosis indicative of neurologic morbidity by 1 year of age (see footnote of Supplementary Table 1 for frequency of specific diagnoses within the MIA group and footnote of Supplementary Table 2 for neurologic morbidities). Women with MIA had significantly increased odds of giving birth at earlier gestational ages (GA) when compared to women without MIA (OR: 6.74; 95% CI: 1.61–28.17, OR: 6.34; 95% CI: 3.37–11.94, & OR: 3.17; 95% CI: 1.72–5.84 for <25 weeks GA, 25–31 weeks GA, & 32–36 weeks GA, respectively). Other demographic and characteristic factors were similar between women with MIA and those without (Table 1). All 65 maternal serum biomarkers exhibited similar concentrations between women with MIA and those without (Supplementary Table 1).

**Table 1:** Characteristics of mothers with and without clinical diagnosis suggestive of MIA.

Sample	MIA present (*n* = 80)	MIA absent (*n* = 522)	Odds ratio (95% CI)	*P*-Value
Race/ethnicity				
White non-Hispanic	30 (37.5)	172 (33.0)	Reference	Reference
Hispanic	32 (40.0)	234 (44.8)	0.78 (0.46–1.34)	0.3733
Black	3 (3.8)	10 (1.9)	1.72 (0.45–6.62)	0.4301
Asian	9 (11.3)	83 (15.9)	0.62 (0.28–1.37)	0.2381
Other	6 (7.5)	23 (4.4)	1.50 (0.56–3.98)	0.4201
Maternal age				
<18 years	1 (1.3)	8 (1.5)	0.84 (0.10–6.81)	0.8673
18–34 years	58 (72.5)	388 (74.3)	Reference	Reference
>34 years	21 (26.3)	126 (24.1)	1.12 (0.65–1.91)	0.6919
Maternal education				
<12 years	12 (15.0)	96 (18.4)	0.65 (0.30–1.43)	0.2861
12 years	18 (22.5)	94 (18.0)	Reference	Reference
>12 years	45 (56.3)	292 (55.9)	0.81 (0.44–1.46)	0.4734
Unknown	5 (6.3)	40 (7.7)	0.65 (0.23–1.88)	0.4292
Gestational age at delivery				
< 25 weeks	3 (3.8)	7 (1.3)	6.74 (1.61–28.2)	0.0089
25–31 weeks	29 (36.3)	72 (13.8)	6.34 (3.37–11.9)	<0.0001
32–36 weeks	29 (36.3)	144 (27.6)	3.17 (1.72–5.84)	0.0002
37–42 weeks	19 (33.8)	299 (57.3)	Reference	Reference
>42 weeks	0 (0.0)	0 (0.0)	NA	NA
Trimester prenatal care began				
1st	77 (96.3)	502 (96.2)	Reference	Reference
2nd	2 (2.5)	15 (2.9)	0.87 (0.20–3.88)	0.8542
3rd	0 (0.0)	1 (0.2)	NA	0.9872
Unknown	1 (1.3)	4 (0.8)	1.63 (0.18–14.7)	0.664
Adequacy of prenatal care				
Adequate	70 (87.5)	475 (91.0)	Reference	Reference
Intermediate	3 (3.8)	24 (4.6)	0.85 (0.25–2.89)	0.7924
Inadequate	2 (2.5)	7 (1.3)	1.94 (0.40–9.52)	0.4148
Unknown	5 (6.3)	16 (3.1)	2.12 (0.75–5.97)	0.1546
Payment for delivery				
Private insurance	41 (51.3)	289 (55.4)	Reference	Reference
Public insurance	37 (46.3)	222 (42.5)	1.18 (0.73–1.89)	0.5086
Other	2 (2.5)	11 (2.1)	1.28 (0.27–5.99)	0.7524
WIC participation				
No	40 (50.0)	292 (55.9)	Reference	Reference
Yes	38 (47.5)	227 (43.5)	1.22 (0.76–1.97)	0.4097
Unknown	2 (2.5)	3 (0.6)	4.87 (0.79–30.0)	0.0882
Infant sex				
Male	38 (47.5)	276 (52.9)	Reference	
Female	42 (52.5)	246 (47.1)	1.24 (0.77–1.99)	0.3709
Prepregnancy BMI (kg/m^2^)				
<18.5	5 (6.3)	25 (4.8)	1.90 (0.68–5.33)	0.2225
18.5–24.9	30 (37.5)	285 (54.6)	Reference	Reference
25–29.9	17 (21.3)	110 (21.1)	1.47 (0.78–2.77)	0.2354
≥30.0	28 (35.0)	102 (19.5)	2.61 (1.49–4.58)	0.0008
Smoked				
No	79 (98.8)	514 (98.5)	Reference	Reference
Yes	1 (1.3)	8 (1.5)	0.81 (0.10–6.59)	0.8466
Parity				
Nulliparous	38 (47.5)	226 (43.3)	Reference	Reference
Multiparous	42 (52.5)	296 (56.7)	0.84 (0.53–1.35)	0.4807
Interpregnancy interval				
<6 months	2 (2.5)	27 (5.2)	0.41 (0.09–1.89)	0.2494
6–23 months	10 (12.5)	98 (18.8)	0.56 (0.24–1.31)	0.1795
24–59 months	15 (18.8)	82 (15.7)	Reference	Reference
>59 months	8 (10.0)	51 (9.8)	0.86 (0.34–2.17)	0.7451
Nulliparous	38 (47.5)	226 (43.3)	0.92 (0.48–1.76)	0.7991
Unknown	7 (8.8)	38 (7.3)	1.01 (0.38–2.67)	0.9888

The multivariable model for prediction of MIA included two characteristics (body mass index (BMI) and GA), three biomarkers included as continuous factors [soluble Fas ligand (sFasL), TNF-related apoptosis-inducing ligand (TRAIL), Adiponectin] and four biomarkers included as categorical factors by low/high quartile [macrophage colony-stimulating factor (M-CSF), Macrophage Inflammatory Proteins 1B (MIP1B), transforming growth-factor-alpha (TGFa), and Leptin]. Variables that increased the odds of an MIA diagnosis included BMI ranges outside of 18.5–24.9, gestational age <37 weeks (by grouping), increased levels of sFASL, TRAIL, and adiponectin, low levels of M-CSF, and both high and low levels of TGFa. Lower odds of MIA diagnosis were associated with high levels of M-CSF and both high and low levels of MIP1B and Leptin (Table 2). Overall, the MIA maternal characteristic plus biomarker model demonstrated good performance with an AUC of 0.814 (95% CI: 0.763–0.865) (with significantly better performance than models containing only biomarkers (AUC: 0.731, 95% CI: 0.669–0.793) or only BMI and gestational age (AUC: 0.728, 95% CI: 0.670–0.786)) (Supplementary Table 3). Cross-validation results were similar to the originally built models (AUC 0.765, 95% CI: 0.709–0.821).

**Table 2:** Model build performed for diagnosis indicative of MIA.

Variable	Parameter estimate	Standard error	Odds ratio (95% CI)	*P*-value
Intercept	−17.18	3.16		<0.0001
BMI				
<18.5	1.00	0.58	2.71 (0.87–8.49)	0.0871
25–29.9	0.47	0.36	1.61 (0.79–3.26)	0.1916
≥30.0	1.14	0.33	3.14 (1.66–5.95)	0.0004
Gestational age				
<25 weeks	2.22	0.80	9.17 (1.93–43.68)	0.0054
25–31 weeks	1.96	0.35	7.08 (3.54–14.13)	<0.0001
32–36 weeks	1.37	0.34	3.92 (2.00–7.70)	<0.0001
sFASL	0.96	0.35	2.61 (1.31–5.20)	0.0062
TRAIL	1.31	0.44	3.70 (1.56–8.78)	0.0030
Adiponectin	0.28	0.07	1.32 (1.14–1.53)	0.0002
M-CSF				
<25th percentile	1.49	0.48	4.44 (1.73–11.42)	0.0019
>75th percentile	−1.37	0.51	0.26 (0.09–0.69)	0.0072
MIP1B				
<25th percentile	−0.88	0.45	0.42 (0.17–1.00)	0.0498
>75th percentile	−0.77	0.38	0.46 (0.22–0.97)	0.0403
TGFa				
<25th percentile	1.93	0.50	6.91 (2.59–18.47)	0.0001
>75th percentile	1.02	0.50	2.77 (1.05–7.33)	0.0397
Leptin				
<25th percentile	−1.17	0.40	0.31 (0.14–0.69)	0.0038
>75th percentile	−0.40	0.34	0.67 (0.35–1.29)	0.2288

AUC 0.814 (0.763–0.865).

References for categorical variables: BMI - normal; Gestational Age - 37–42 weeks; M-CSF, MIP1B, TGFa, Leptin - 25–75th percentile. All biomarkers natural log transformed.

Infants born to a mother with a diagnosis indicative of MIA were more than twice as likely (OR 2.38, 95% CI: 1.26–4.52) to experience a neurologic morbidity than were infants born to a mother without MIA. While there were no significant crude differences in maternal serum biomarkers among infants with a neurologic morbidity and those without (Supplementary Table 2), we observed significant differences in adjusted models for neurologic morbidity (AUC = 0.802, 95% CI: 0.747–0.856). Within the final model, gestational age <37 weeks (by grouping), insurance status, and high and low levels of Interleukin 12 p70 (IL12p70) and Macrophage Inflammatory Proteins 1A (MIP1A) were associated with greater risk of neurologic morbidity. Lower levels of Vascular endothelial growth-factor receptor 3 (VEGFR3) and high and low levels of Interleukin 5 (IL-5) and Interleukin 15 (IL-15) were associated with lower risk of neurologic morbidity (Table 3). MIA clinical diagnosis did not enter the model independently but when forced into the model, the AUC increased to 0.808 (95% CI: 0.757–0.860) (Supplementary Table 4). Cross-validation results were similar to the originally built models (AUC 0.748, 95% CI: 0.687–0.809).

**Table 3:** Model build performed for infant neurologic morbidity without interference.

Variable	Parameter estimate	Standard error	Odds ratio (95% CI)	*P*-value
Intercept	−2.10	0.68	NA	0.002
Insurance				
Medi-Cal	0.38	0.3	1.47 (0.81–2.66)	0.2088
Other	1.85	0.75	6.38 (1.48–27.48)	0.0129
Gestational age				
<25 weeks	2.9	0.77	18.24 (4.05–82.16)	0.0002
25–31 weeks	1.95	0.39	6.99 (3.24–15.09)	<0.0001
32–36 weeks	1.15	0.38	3.17 (1.51–6.65)	0.0023
VEGFR3	−0.27	0.12	0.76 (0.61–0.96)	0.0201
IL12p70				
<25th percentile	1.66	0.53	5.24 (1.86–14.78)	0.0018
>75th percentile	1.36	0.62	3.9 (1.17–13.05)	0.0272
IL-15				
<25th percentile	−0.9	0.49	0.41 (0.16–1.06)	0.0664
>75th percentile	−1.34	0.54	0.26 (0.09–0.76)	0.0139
IL-5				
<25th percentile	−1.5	0.52	0.22 (0.08–0.62)	0.0038
>75th percentile	−1.5	0.59	0.22 (0.07–0.70)	0.0104
MIP1A				
<25th percentile	1.39	0.41	4.01 (1.80–8.90)	0.0007
>75th percentile	0.22	0.42	1.25 (0.54–2.86)	0.6025

AUC 0.802 (0.747–0.856).

References for categorical variables: Insurance - private; Gestational age - 37–42 weeks; M-IL12p70, IL-15, IL-5, MIP1A - 25–75th percentile; All biomarkers natural log transformed.

## Discussion

Results from our study show that MIA is related to several prenatal clinical and immune factors, including BMI > 29.9 and abnormal levels of sFASL, TRAIL, adiponectin, M-CSF, and TGFa levels. Our analyses also identified several pre- and postnatal factors to be associated with neurologic morbidity in infants including lower gestational age, insurance type, and differences in circulating prenatal cytokines (IL12p70, MIP1A, IL-5, IL-15).

MIA has been previously defined as an immunologic alteration triggered by heterogeneous inflammatory stimuli during pregnancy like infection, autoimmune disorders, allergy, asthma exacerbation, malignancies etc. Our study aimed to explore the biochemical and demographic profile of pregnant women to objectively identify mothers with immune activation during pregnancy. Amongst the biochemical factors found to be associated with MIA in the current study, sFasL (also CD95L) and TRAIL, both belonging to the TNF family, are known to play pivotal roles in trophoblastic immune privilege during pregnancy, facilitating maternal immune tolerance and trophoblast invasion [29]. A small number of studies have also noted dysregulation of FASL and TRAIL levels to be associated with pre-eclampsia [28, 30, 31], fetal growth restriction [31] and preterm birth [32]. Similarly, abnormal levels of M-CSF have been linked with recurrent fetal loss and pre-eclampsia in some studies [33, 34]. M-CSF is known to be a critical trophic factor for microglia survival and differentiation which is of paramount importance for normal fetal brain development [35, 36]. As such, derangement of M-CSF during pregnancy has the potential to disrupt multiple processes vital for normal fetal brain development including neural cell survival and neural network formation [35, 37, 38]. Importantly, to the best of our knowledge, our study is the first to comprehensively characterize the immune and biochemical profile of women with MIA.

Further, we found an increased risk of neurologic morbidity within the first year of life in infants born to mothers with differing levels of pro-inflammatory cytokines including IL12p70 and MIP1A. These findings support previous animal and epidemiologic studies identifying several maternal pro-inflammatory cytokines as critical mediators of MIA and secondary disease phenotypes in offspring [9, 19–23, 39]. In animal models, a single injection of an inflammatory cytokine (interleukin (IL)-6, -17, or -2) induces MIA and is sufficient to result in ASD and SZ-like behaviors in offspring [9, 19]. Moreover, co-administration of Poly I:C with an IL-6 or an IL-17 function blocking antibody, or overexpression of the anti-inflammatory cytokine IL-10, partially prevents the effects of MIA in offspring [9, 17]. Examination of cytokines and chemokines in archived maternal serum samples in the Early Markers for Autism (EMA) study demonstrated that elevated levels of GM-CSF, interferon-γ, IL-1α, and IL-6 were associated with autism spectrum disorder with intellectual disability [22]. Conversely, significantly lower levels of the chemokines IL-8 and MCP-1 in maternal serum were associated with developmental delay in children [10]. Specifically, in amniotic fluid samples from a Danish study, several cytokines, including TNF-α, IL-4, and IL-10 were related to increased risk of ASD in children [21]. In another study from the EMA group, they found that elevated levels of IL-4, IL-8, and IL-12p70 in neonatal blood samples were associated with ASD [23].

In addition to identifying factors associated with an increased risk of neurologic morbidity, our study also highlights factors that promote resilience against this morbidity. Our analyses suggest that IL-5 and IL-15 may impart a protective effect on the developing brain of the fetus. IL-5 is a pleotropic cytokine that regulates atopic reactions, and it is required for neuronal differentiation of hippocampal progenitors during neurodevelopment [40]. In contrast to our findings, Goines et al. reported elevated levels of IL-5 to be associated with increased risk of ASD [24]. IL-15 also exerts pleotropic functions with low concentrations of IL-15 favoring anti-inflammatory IL-10 production [41]. Additionally, IL-15 regulates neural stem cells proliferation during neurodevelopment [42]. High concentrations of IL-15, however, favor TNF-α, IL-1, and IL-6 production, which have been associated with poor neurodevelopmental outcomes [43].

Results from the present study also suggested that women with BMI > 29.9 kg/m^2^ are more vulnerable to immune activation which is in line with previous studies showing chronic immune activation in obese individuals [44, 45]. Additionally, previous epidemiologic studies provide evidence that maternal obesity and metabolic complications increase the risk of autism spectrum disorders, anxiety, depression, schizophrenia, and impairments in cognition in offspring [46–49]. However, the mechanisms underlying this association are not fully understood. Based on our results, maternal obesity might represent a pre-existing inflammatory state which can interact with acute inflammatory stimuli during pregnancy resulting in adverse outcomes in the offspring. Further exploration of the cytokine profile in obese individuals may shed light on its role within the MIA spectrum and complex interactions between these different risk factors. In the neurologic morbidity model, insurance status was also a significant factor in increasing neurologic morbidity in infants. We suspect that in our model, having public insurance is serving as a proxy for unmeasured or underreported factors associated with poor neurologic outcomes including, possibly, the lack of access to proper nutrition, increased psycho-social or systemic stress and discrimination, and greater exposure to potentially harmful substances with close links to poverty (e.g., substance exposure, pollution). Lastly, as expected, lower gestational age was related with increased risk of neurologic morbidity in infants. Low gestational age, obesity, and poverty have been associated with adverse neonatal outcomes in previous studies [50, 51]. In our study, built a vulnerability profile that includes both maternal demographic and biochemical parameters to identify pregnancies most susceptible to infant neurologic morbidity. While the association between each of those factors and adverse outcomes is known, our study examined how a combination of these factors, along with immune biomarkers, can enhance the identification of at-risk infants in a more objective manner. By analyzing a wide range of variables simultaneously, we hope to provide a more comprehensive understanding of the complex interplay between maternal factors and neonatal outcomes.

Overall, the results from our study support the previously described hypothesis that pro-inflammatory cytokines may significantly alter the intra-uterine environment and thereby disrupt the neurodevelopmental process in the fetus [7, 17, 36]. Although, we did not identify the more commonly studied cytokines and chemokines in animal models of MIA – IL-6, IL-17, TNF-α as being associated with neurologic morbidity in our study. Possible explanations for these differences range from phenotypic heterogeneity, a polygenic etiology, a difference in time from inflammatory exposure to sample collection, and differences in detection sensitivity of different assay panels and potential lack of statistical power. It is important that future studies follow-up on these heterogeneous study findings.

During a healthy pregnancy, the maternal immune system undergoes significant changes that allow for the maintenance of an immune environment characterized by a unique state of tolerance that avoids rejection of the fetal-placental unit [27]. Data suggests that disturbance of this highly regulated immune balance can dramatically compromise placental integrity and lead to trans-placental transfer of these cytokines, which can directly disrupt the developing fetal brain [7, 17, 36]. Downstream activation of the placental immune cells by the circulating pro-inflammatory cytokines can detrimentally alter the intra-uterine environment too leading to a disruption of neurological development in the fetus [52, 53]. Notably, our data support and further the findings of others that suggest the alteration of the intra-uterine environment secondary to MIA may lead to a chronic state of inflammation and neural disruption in the offspring [7, 17, 52, 53]. Our data also support the findings of others that suggest that an anti-inflammatory response in mothers and adequate nutritional and metabolic status characterized by a normal BMI may promote neurodevelopmental resilience in infants exposed to MIA.

Our study adds to the existing literature by utilizing predictive models including maternal demographic and clinical characteristics, as well as immune and growth-factor markers to identify vulnerability and resilience factors associated with MIA and neurologic morbidity in infants. With the knowledge gained from this study, healthcare providers can develop tailored interventions for pregnant women at higher risk. For example, women with obesity and low SES might benefit from additional monitoring, nutritional support, or interventions aimed at reducing inflammation during pregnancy. Healthcare resources can be allocated more efficiently to closely monitor at-risk infants in neonatal intensive care units (NICUs) and enhance access to early intervention services. Healthcare providers can use the study’s findings to educate pregnant women about the importance of factors like maintaining a healthy BMI and managing stress during pregnancy.

While the present study points to risk and resilience factors for MIA and associated neurologic morbidity in children, there are important limitations of these analyses inherent to a retrospective registry-based study. Most notably, the study sample was a retrospective cohort and thus not powered to detect all differences in neurologic morbidity in infants. It is also important to note that because this study relied on women who had already elected to participate in prenatal screening for aneuploidies and neural tube defects, this sample is biased towards women who made that choice and as such, it is unclear if performance would generalize to all pregnant women. The biological specimens evaluated were maternal serum samples and may not reflect key intricacies in the intra-uterine environment or the immune changes in the fetus itself. Additionally, the serum biomarkers were measured at 15–20 weeks of gestation as part of routine prenatal screening. While this timing allows for the assessment of immune markers during mid-pregnancy, it may not capture changes that occur later in pregnancy. Also, our outcome measure included neurodevelopmental morbidity up to 1 year of age based on electronic medical records from hospital visits and did not include data from outpatient clinics. As such, we potentially underestimated the prevalence of neurodevelopmental morbidity in our study population. This highlights the need for future prospective investigations to comprehensively assess neurologic morbidities associated with the specified prenatal risk factors over extended periods within a consistent outpatient setting.

In conclusion, we show that cytokine constellations, measured by a single multiplex assay, considered in combination with demographic and clinical characteristics, are closely associated with MIA and with neurological outcomes in infants. Our findings show that mid-pregnancy inflammatory environment, characterized by elevated pro-inflammatory and diminished anti-inflammatory cytokine levels in pregnant women with pre-existing risk factors like high BMI and low SES, could render their children more susceptible to neurologic morbidity. Thus, pointing to early life immune-related factors that are not only pathophysiologically important, but may also be modifiable. Early identification of these high-risk pregnancies could potentially allow for the implementation of preventative strategies aimed at decreasing inflammation. Further research into downstream cellular and molecular impact of factors like IL-12p70, MIP1A, IL-4, IL-6, IL-8, and IL-17 is necessary to understand specific mechanisms and to develop therapeutic interventions.

### Supplementary information

Supplemental tables

## Data Availability

The data use agreement with the California Department of Health Care Access and Information prohibits distribution of any patient-level data; thus, the data used for this study are not made publicly available. Data can be requested from Department of Health Care Access and Information (https://hcai.ca.gov/) by qualified researchers.
