# Supplementary material for: Assessing for prenatal risk factors associated with infant neurologic morbidity using a multivariate analysis
Source: J Perinatol. 2023 Nov 10;43(12):1486–93. doi: 10.1038/s41372-023-01820-3 (PMC10716040; doi:10.1038/s41372-023-01820-3)
Supplement: Supplementary file 1 — Supplemental tables [file 41372_2023_1820_MOESM1_ESM.docx]

Supplementary table 1. Serum biomarkers in women with and without diagnoses suggestive of MIA^a^

| **Serum Biomarkers** | **MIA Present (n=80)** | **MIA Absent (n=523)** | **P-Value** |
| --- | --- | --- | --- |
| **Interleukins** |  |  |  |
| IL-1A | 4.21 (4.00-4.43) | 4.20 (4.13-4.28) | 0.9358 |
| IL-1RA | 5.61 (5.47-5.75) | 5.60 (5.54-5.65) | 0.8769 |
| IL-1R1 | 3.01 (2.74-3.27) | 2.96 (2.86-3.07) | 0.7718 |
| IL-1R2 | 6.80 (6.57-7.04) | 6.81 (6.72-6.90) | 0.9658 |
| IL-1B | 3.34 (2.86-3.82) | 3.37 (3.18-3.55) | 0.9207 |
| IL-2 | 4.26 (4.02-4.49) | 4.23 (4.14-4.32) | 0.8266 |
| IL-2RA | 5.64 (5.52-5.76) | 5.60 (5.55-5.65) | 0.4815 |
| IL-4 | 3.65 (3.32-3.99) | 3.62 (3.50-3.75) | 0.8578 |
| IL-4R | 4.54 (4.38-4.70) | 4.60 (4.54-4.65) | 0.4677 |
| Il-5 | 4.95 (4.86-5.05) | 4.93 (4.89-4.97) | 0.6864 |
| IL-6 | 4.60 (4.45-4.74) | 4.53 (4.46-4.60) | 0.4873 |
| IL6R | 9.26 (9.16-9.35) | 9.29 (9.26-9.32) | 0.4466 |
| GP130 | 10.25 (9.93-10.57) | 10.29 (10.17-10.42) | 0.8074 |
| IL-7 | 5.51 (5.42-5.60) | 5.50 (5.46-5.54) | 0.9026 |
| IL-10 | 4.10 (3.84-4.36) | 4.03 (3.93-4.13) | 0.6494 |
| IL-12p40 | 4.79 (4.40-5.19) | 4.80 (4.66-4.93) | 0.9905 |
| IL-12p70 | 4.82 (4.72-4.91) | 4.76 (4.72-4.80) | 0.3461 |
| IL-13 | 3.68 (3.50-3.86) | 3.64 (3.57-3.71) | 0.6858 |
| IL-15 | 3.86 (3.66-4.06) | 3.86 (3.79-3.94) | 0.9856 |
| IL-17 | 4.19 (3.97-4.42) | 4.11 (4.02-4.19) | 0.4439 |
| IL17F | 2.00 (1.37-2.64) | 2.07 (1.85-2.29) | 0.8224 |
| **Interferons** |  |  |  |
| IFNA | 3.38 (2.97-3.80) | 3.22 (3.05-3.38) | 0.4708 |
| IFNB | 2.73 (2.28-3.18) | 2.70 (2.53-2.86) | 0.8815 |
| IFNG | 2.79 (2.27-3.31) | 2.62 (2.41-2.83) | 0.5561 |
| **Chemokine Ligands** |  |  |  |
| MCP1 | 4.24 (3.84-4.64) | 4.15 (4.01-4.30) | 0.6639 |
| MIP1A | 3.65 (3.25-4.05) | 3.61 (3.47-3.76) | 0.8588 |
| MIP1B | 5.11 (4.96-5.26) | 5.07 (5.01-5.12) | 0.5996 |
| RANTES | 8.65 (8.42-8.88) | 8.67 (8.58-8.76) | 0.8489 |
| MCP3 | 4.11 (3.83-4.38) | 4.09 (3.99-4.19) | 0.9063 |
| Eotaxin | 3.85 (3.55-4.14) | 3.82 (3.71-3.93) | 0.8512 |
| GRO-A | 3.43 (3.02-3.84) | 3.28 (3.11-3.45) | 0.5228 |
| ENA-78 | 4.95 (4.77-5.13) | 4.75 (4.69-4.82) | 0.0309 |
| IL-8 | 5.83 (5.33-6.33) | 5.83 (5.65-6.01) | 0.9929 |
| MIG | 4.56 (4.26-4.87) | 4.39 (4.26-4.52) | 0.3461 |
| IP-10 | 4.53 (4.34-4.72) | 4.47 (4.40-4.54) | 0.5473 |
| **TNF Alpha Super Family** |  |  |  |
| TNFA | 2.88 (2.47-3.29) | 2.78 (2.61-2.94) | 0.6476 |
| TNFR1 | 6.25 (6.15-6.35) | 6.20 (6.16-6.24) | 0.3661 |
| TNFR2 | 8.24 (8.16-8.33) | 8.23 (8.20-8.26) | 0.8257 |
| CD30 | 2.99 (2.82-3.16) | 2.96 (2.89-3.03) | 0.7768 |
| CD40L | 5.73 (5.51-5.95) | 5.77 (5.68-5.85) | 0.7554 |
| sFASL | 4.49 (4.20-4.78) | 4.38 (4.27-4.49) | 0.4641 |
| TNFB | 4.12 (3.76-4.48) | 4.02 (3.88-4.17) | 0.6219 |
| TRAIL | 4.47 (4.29-4.64) | 4.40 (4.34-4.46) | 0.4665 |
| **Growth Factors** |  |  |  |
| TGFA | 4.01 (3.72-4.29) | 3.93 (3.83-4.04) | 0.6045 |
| TGFB | 3.26 (2.96-3.56) | 3.29 (3.18-3.40) | 0.8611 |
| SCF | 4.40 (4.16-4.63) | 4.33 (4.24-4.42) | 0.5769 |
| LIF | 3.46 (3.13-3.78) | 3.43 (3.30-3.55) | 0.8426 |
| PDGFBB | 6.42 (6.28-6.57) | 6.42 (6.36-6.48) | 0.9592 |
| FGF-Basic | 4.78 (4.62-4.94) | 4.73 (4.67-4.80) | 0.5862 |
| NGF | 3.95 (3.86-4.03) | 3.94 (3.91-3.97) | 0.8569 |
| VEGF | 4.10 (3.92-4.29) | 4.11 (4.04-4.17) | 0.9771 |
| VEGFR1 | 6.74 (6.18-7.31) | 6.86 (6.64-7.07) | 0.7094 |
| VEGFR2 | 7.51 (7.13-7.89) | 7.50 (7.35-7.65) | 0.9456 |
| VEGFR3 | 5.23 (4.86-5.60) | 5.14 (5.00-5.28) | 0.6477 |
| HGF | 4.71 (4.45-4.97) | 4.69 (4.59-4.78) | 0.8752 |
| **Colony Stimulating Factors** |  |  |  |
| G-CSF | 2.86 (2.60-3.12) | 2.71 (2.58-2.83) | 0.3703 |
| GM-CSF | 4.23 (4.05-4.42) | 4.25 (4.18-4.32) | 0.8987 |
| M-CSF | 4.34 (4.06-4.62) | 4.31 (4.21-4.42) | 0.8697 |
| **Soluble Adhesion Molecules** |  |  |  |
| sICAM1 | 9.15 (8.90-9.39) | 9.04 (8.95-9.13) | 0.3855 |
| sVCAM1 | 9.40 (9.11-9.68) | 9.31 (9.22-9.40) | 0.5271 |
| **Others** |  |  |  |
| Leptin | 7.95 (7.77-8.13) | 7.88 (7.80-7.96) | 0.4959 |
| PAI1 | 7.29 (7.09-7.48) | 7.27 (7.20-7.35) | 0.9029 |
| Resistin | 7.90 (7.76-8.05) | 7.83 (7.78-7.89) | 0.3360 |
| RAGE | 3.72 (3.55-3.90) | 3.66 (3.59-3.72) | 0.4807 |
| Adiponectin | 12.89 (11.93-13.85) | 12.72 (12.35-13.09) | 0.7368 |

All biomarkers natural log transformed.

^a^Diagnoses in the MIA grouping included women with: Infection n=29, Autoimmune disorder n=19, Allergy n= 2, Asthma n=22 and Malignancy n=17

Supplementary table 2. Maternal serum biomarkers in Infants with and without neurologic morbidity^a^

| **Serum Biomarkers** | **Neurologic Morbidity Present (n=61)** | **Neurologic Morbidity Absent (n=541)** | **P-Value** |
| --- | --- | --- | --- |
| **Interleukins** |  |  |  |
| IL-1A | 4.08 (3.85-4.30) | 4.22 (4.14-4.30) | 0.2331 |
| IL-1RA | 5.51 (5.36-5.65) | 5.61 (5.56-5.66) | 0.2299 |
| IL-1R1 | 2.98 (2.70-3.27) | 2.97 (2.87-3.07) | 0.9340 |
| IL-1R2 | 6.87 (6.58-7.15) | 6.80 (6.71-6.89) | 0.6594 |
| IL-1B | 3.31 (2.74-3.88) | 3.37 (3.19-3.55) | 0.8327 |
| IL-2 | 4.17 (3.91-4.42) | 4.24 (4.15-4.33) | 0.6078 |
| IL-2RA | 5.64 (5.49-5.78) | 5.60 (5.55-5.65) | 0.5927 |
| IL-4 | 3.55 (3.19-3.92) | 3.64 (3.51-3.76) | 0.6713 |
| IL-4R | 4.56 (4.37-4.74) | 4.59 (4.54-4.64) | 0.7001 |
| Il-5 | 4.88 (4.77-4.99) | 4.94 (4.90-4.98) | 0.3589 |
| IL-6 | 4.49 (4.33-4.65) | 4.54 (4.47-4.61) | 0.6080 |
| IL6R | 9.35 (9.24-9.46) | 9.28 (9.25-9.31) | 0.1682 |
| GP130 | 10.31 (9.95-10.68) | 10.29 (10.17-10.41) | 0.8909 |
| IL-7 | 5.45 (5.34-5.56) | 5.51 (5.47-5.55) | 0.3530 |
| IL-10 | 3.93 (3.63-4.23) | 4.06 (3.96-4.15) | 0.4130 |
| IL-12p40 | 4.54 (4.08-4.99) | 4.82 (4.69-4.96) | 0.1888 |
| IL-12p70 | 4.71 (4.60-4.82) | 4.78 (4.74-4.82) | 0.2692 |
| IL-13 | 3.58 (3.37-3.78) | 3.65 (3.58-3.73) | 0.4985 |
| IL-15 | 3.77 (3.56-3.98) | 3.87 (3.80-3.95) | 0.3821 |
| IL-17 | 4.03 (3.79-4.28) | 4.13 (4.05-4.21) | 0.4598 |
| IL17F | 1.85 (1.14-2.56) | 2.09 (1.87-2.31) | 0.4953 |
| **Interferons** |  |  |  |
| IFNA | 2.87 (2.32-3.41) | 3.28 (3.12-3.44) | 0.1132 |
| IFNB | 2.56 (2.06-3.07) | 2.72 (2.56-2.87) | 0.5463 |
| IFNG | 2.48 (1.84-3.13) | 2.66 (2.46-2.86) | 0.5868 |
| **Chemokine Ligands** |  |  |  |
| MCP1 | 4.09 (3.67-4.50) | 4.17 (4.03-4.32) | 0.7056 |
| MIP1A | 3.24 (2.76-3.72) | 3.66 (3.52-3.80) | 0.0674 |
| MIP1B | 4.96 (4.82-5.10) | 5.08 (5.03-5.14) | 0.1646 |
| RANTES | 8.64 (8.37-8.91) | 8.67 (8.59-8.76) | 0.8010 |
| MCP3 | 4.01 (3.71-4.31) | 4.10 (4.00-4.20) | 0.5636 |
| Eotaxin | 3.75 (3.42-4.07) | 3.83 (3.72-3.94) | 0.6390 |
| GRO-A | 3.25 (2.75-3.75) | 3.31 (3.14-3.47) | 0.8254 |
| ENA-78 | 4.78 (4.59-4.97) | 4.78 (4.71-4.84) | 0.9979 |
| IL-8 | 5.81 (5.22-6.39) | 5.83 (5.66-6.01) | 0.9261 |
| MIG | 4.31 (3.90-4.71) | 4.43 (4.30-4.56) | 0.5471 |
| IP-10 | 4.40 (4.20-4.59) | 4.49 (4.42-4.56) | 0.3950 |
| **TNF Alpha Super Family** |  |  |  |
| TNFA | 2.71 (2.23-3.18) | 2.80 (2.64-2.96) | 0.7116 |
| TNFR1 | 6.24 (6.13-6.36) | 6.20 (6.16-6.24) | 0.5367 |
| TNFR2 | 8.24 (8.13-8.35) | 8.23 (8.20-8.26) | 0.8823 |
| CD30 | 2.88 (2.68-3.08) | 2.98 (2.91-3.04) | 0.3701 |
| CD40L | 5.64 (5.42-5.87) | 5.78 (5.69-5.86) | 0.3109 |
| sFASL | 4.31 (3.96-4.65) | 4.41 (4.30-4.51) | 0.5605 |
| TNFB | 3.93 (3.44-4.41) | 4.05 (3.91-4.19) | 0.5888 |
| TRAIL | 4.37 (4.19-4.55) | 4.42 (4.36-4.48) | 0.6146 |
| **Growth Factors** |  |  |  |
| TGFA | 3.82 (3.52-4.12) | 3.96 (3.85-4.06) | 0.4046 |
| TGFB | 3.25 (2.91-3.58) | 3.29 (3.18-3.40) | 0.8163 |
| SCF | 4.28 (4.02-4.55) | 4.34 (4.26-4.43) | 0.6706 |
| LIF | 3.38 (3.02-3.73) | 3.44 (3.31-3.56) | 0.7657 |
| PDGFBB | 6.42 (6.22-6.61) | 6.42 (6.36-6.48) | 0.9572 |
| FGF-Basic | 4.69 (4.52-4.87) | 4.74 (4.68-4.81) | 0.5993 |
| NGF | 3.87 (3.78-3.95) | 3.95 (3.92-3.98) | 0.0814 |
| VEGF | 4.05 (3.86-4.24) | 4.11 (4.05-4.18) | 0.5470 |
| VEGFR1 | 6.86 (6.22-7.50) | 6.84 (6.62-7.05) | 0.9433 |
| VEGFR2 | 7.52 (7.08-7.97) | 7.50 (7.35-7.64) | 0.9149 |
| VEGFR3 | 5.00 (4.57-5.42) | 5.17 (5.04-5.31) | 0.4212 |
| HGF | 4.61 (4.32-4.91) | 4.70 (4.60-4.79) | 0.5750 |
| **Colony Stimulating Factors** |  |  |  |
| G-CSF | 2.67 (2.34-3.01) | 2.73 (2.61-2.85) | 0.7614 |
| GM-CSF | 4.18 (3.99-4.37) | 4.25 (4.18-4.32) | 0.4866 |
| M-CSF | 4.28 (3.96-4.60) | 4.32 (4.22-4.43) | 0.7968 |
| **Soluble Adhesion Molecules** |  |  |  |
| sICAM1 | 8.86 (8.63-9.10) | 9.07 (8.99-9.16) | 0.1313 |
| sVCAM1 | 9.23 (8.93-9.52) | 9.33 (9.24-9.43) | 0.4598 |
| **Others** |  |  |  |
| Leptin | 7.94 (7.65-8.24) | 7.88 (7.81-7.95) | 0.6029 |
| PAI1 | 7.25 (7.02-7.48) | 7.28 (7.21-7.35) | 0.7884 |
| Resistin | 7.80 (7.64-7.96) | 7.85 (7.80-7.90) | 0.5335 |
| RAGE | 3.58 (3.35-3.81) | 3.68 (3.61-3.74) | 0.3554 |
| Adiponectin | 12.58 (11.51-13.65) | 12.76 (12.4-13.12) | 0.7571 |

All biomarkers natural log transformed.

^a^Diagnoses included in the neurologic morbidity grouping included infants with: PVL n=1, Convulsions/seizures n=4, Abnormal neuro imaging n=6, Abnormal neuro exam n=54, IVH n=0

Supplementary table 3. Comparison of characteristics only, biomarkers only, and combined model for MIA.

| **Model** | **AUC (95% confidence interval)** | **P-Value** |
| --- | --- | --- |
| **BMI & Gestational age only** | 0.728 (0.670-0.786) | 0.0006 |
| **Serum biomarkers only** | 0.731 (0.669-0.793) | 0.0013 |
| **Both** | 0.814 (0.763-0.865) | Reference |

Supplementary table 4. Model build performed with MIA forced in at the end. AUC 0.808 (0.757-0.860)

| Variable | Parameter Estimate | Standard Error | Odds Ratio (95% CI) | P-value |
| --- | --- | --- | --- | --- |
| **Intercept** | -2.12 | 0.68 | NA | 0.0019 |
| **MIA** | 0.70 | 0.38 | 2.02 (0.96-4.22) | 0.0627 |
| **Insurance** |  |  |  |  |
| Medi-Cal | 0.43 | 0.31 | 1.53 (0.84-2.80) | 0.1657 |
| Other | 1.92 | 0.73 | 6.83 (1.63-28.70) | 0.0087 |
| **Gestational Age** |  |  |  |  |
| < 25 weeks | 2.70 | 0.77 | 14.87 (3.29-67.13) | 0.0004 |
| 25- 31 weeks | 1.78 | 0.40 | 5.96 (2.70-13.16) | <0.0001 |
| 32- 36 weeks | 1.07 | 0.38 | 2.92 (1.38-6.18) | 0.005 |
| **VEGFFR3** | -0.29 | 0.12 | 0.75 (0.59-0.95) | 0.0147 |
| **IL12p70** |  |  |  |  |
| <25th percentile | 1.79 | 0.54 | 5.97 (2.08-17.14) | 0.0009 |
| >75th percentile | 1.41 | 0.62 | 4.09 (1.22-13.72) | 0.0228 |
| **IL15** |  |  |  |  |
| <25th percentile | -1.03 | 0.50 | 0.36 (0.14-0.95) | 0.0385 |
| >75th percentile | -1.32 | 0.54 | 0.27 (0.09-0.78) | 0.0151 |
| **IL5** |  |  |  |  |
| <25th percentile | -1.50 | 0.52 | 0.22 (0.08-0.62) | 0.0038 |
| >75th percentile | -1.52 | 0.59 | 0.22 (0.07-0.70) | 0.0101 |
| **MIP1A** |  |  |  |  |
| <25th percentile | 1.45 | 0.41 | 4.26 (1.91-9.52) | 0.0004 |
| >75th percentile | 0.16 | 0.43 | 1.17 (0.51-2.70) | 0.7143 |
| MIA: maternal immune activation; References for categorical variables: Insurance - private; Gestational Age - 37-42 weeks; M-IL12p70, IL15, IL5, MIP1A - 25-75th percentile; All biomarkers natural log transformed. | | | |  |
